# Morningness–Eveningness Preference and Motor Wake–Sleep Inertia in Adolescents

**DOI:** 10.3390/s24237668

**Published:** 2024-11-30

**Authors:** Vincenzo Natale, Alice Andreose, Valeria Bacaro, Sara Giovagnoli, Federica Giudetti, Martina Grimaldi, Lorenzo Tonetti, Elisabetta Crocetti

**Affiliations:** Department of Psychology “Renzo Canestrari”, University of Bologna, Viale Berti Pichat 5, 40127 Bologna, Italy; alice.andreose@hotmail.com (A.A.); valeria.bacaro2@unibo.it (V.B.); sara.giovagnoli@unibo.it (S.G.); federica.giudetti3@unibo.it (F.G.); martinagrimaldi18@gmail.com (M.G.); lorenzo.tonetti2@unibo.it (L.T.); elisabetta.crocetti@unibo.it (E.C.)

**Keywords:** actigraphy, circadian preference, motor sleep inertia, motor wake inertia

## Abstract

The aim of the present work was to analyze possible differences in the wake–sleep and sleep–wake transition in relation to adolescents’ circadian preference using actigraphy. Overall, 729 participants were enrolled in the research and 443 of them wore actigraphs on the non-dominant wrist for at least three nights. According to the reduced Morningness–Eveningness Questionnaire for Children and Adolescents cut-off scores, 61 participants belonged to the evening-type category, while 38 participants belonged to the morning-type. We extracted the motor activity counts, minute-by-minute, during the wake–sleep and sleep–wake transitions, to depict the motor wake inertia and motor sleep inertia, respectively. We adopted the functional linear modeling statistical framework to examine the changes in both transitions according to chronotype. Overall, the results show a significantly higher motor wake inertia and lower motor sleep inertia in morning compared to evening types.

## 1. Introduction

The actigraph is a wearable device to measure the activity–rest cycle. Consisting of a triaxial accelerometer, it measures waves of movement like vibrations and/or how fast the movements are [1]. The data collection process is usually performed over at least several days and often several weeks; this both enables and facilitates the identification of patterns of daytime and nighttime activity and non-activity in order to investigate sleep and circadian activity patterns [2].

The importance of activity measurement (actigraphy) in humans, from childhood to adulthood, is now widely recognized in various research and clinical fields. Having been “snubbed” to a degree in the past, the use of actigraphy has exponentially increased in the last thirty years. For example, a search in any citation database using “actigraphy” as keyword shows that relatively few works were published in 2000 and that by the 2020s the number had risen to several hundred.

This expansion in the use of actigraphy is essentially due to two reasons. The first is technological developments, whereby actigraphs have gone from being rather bulky devices (the first models were worn on the waist or ankle) to smaller ones (wearable on the wrist or finger) capable of recording signals other than motor activity, such as body temperature or heart rate. The second is the change in the use of actigraphy. In the past, actigraphy was mainly used to monitor sleep outside the sleep laboratory for long periods and in an ecological setting. This was possible through the use of algorithms capable of indicating, on the basis of the number of movements, whether a person was awake or sleeping. The guideline set by a task force of experts in sleep medicine [3] provides recommendations for the use of actigraphy in patients with suspected sleep disorders, especially chronic insomnia and circadian rhythm sleep–wake disorders. However, the sleep assessment provided by the actigraph is only an indirect evaluation of sleep and less informative in comparison to polysomnography (PSG), which is capable of showing the sleep architecture, and for this reason is considered the gold standard for sleep studies [4]. For these reasons, in the last few years, researchers have started to focus more on analyzing what actigraphy actually measures, i.e., movement. This interest originated from the desire to study circadian rhythms from a behavioral point of view, therefore depicting motor circadian rhythms. For this purpose, actigraphy is an ideal method since it is ecological (the actigraph is worn by the participant who can continue to freely live outside the laboratory setting), relatively inexpensive, and allows the collection of data for prolonged periods, even months. This new approach has made it possible to extend the use of actigraphy in the clinical setting to other pathologies linked to degenerative problems of the nervous system, such as Alzheimer’s [5] and multiple sclerosis [6].

In this framework, the use of actigraphy was recently proposed in order to study, from a motor point of view, the transition phases from wakefulness to sleep and from sleep to wakefulness. The first phase was labeled motor wake inertia [7], while the second motor sleep inertia [8].

Sleep inertia, a familiar term for somnologists, can be defined as a “transitional state between sleep and wake, marked by impaired performance, reduced vigilance, and a desire to return to sleep” [9] (p. 76). This transition is not based on an all-or-nothing process, but acts according to slow and progressive mechanisms [9]. The sleep inertia phenomenon depends on several factors, such as prior sleep duration [10], sleep stage of awakening [11], and the type of task analyzed as well individual features [12]. It was shown [8] that motor sleep inertia dissipation, ecologically assessed through the recording of motor activity with actigraphy, is completed in 70 min in healthy participants. Conversely, wake inertia corresponds to the transition phase from wakefulness to sleep, therefore corresponding to the falling asleep phase. The greater the wake inertia, the greater the sleep onset latency (SOL). For this reason, wake inertia has been mainly investigated in a clinical setting. The dissipation of motor wake inertia was also examined [7], which was completed around 20 min after bedtime in healthy participants.

The study of motor sleep inertia and motor wake inertia potentially opens up new lines of study both in clinical [13] and basic research [14]. In the present study, we focused in particular on the relationship between motor wake and motor sleep inertia with respect to circadian preference [15]. Individual differences in circadian rhythms essentially refer to the time of falling asleep and waking up. People who spontaneously prefer to get up early and go to bed early are called morning people (or larks). In contrast, people who spontaneously prefer to get up late and go to bed late are called evening people (or owls). Most of the population, around 70%, who fall between these two extremes, are called intermediate [15]. Circadian preference significantly changes with age [16]. During childhood there is a greater preference for morningness, during adolescence there is a progressive shift towards eveningness, while in old age it has been observed that there is a return to a greater preference for morningness.

One of the characteristics that systematically emerges from the questionnaires used to evaluate circadian preference is the so-called “morning affect” [17]. According to Jankosky [18], morning affect refers to the ease with which people wake up in the morning. The term morning affect has been used by some authors as a synonym for morningness [19]. Although morning affect concerns the moment of transition from sleep to wakefulness, it does not represent the same concept of sleep inertia. For example, no correlation has been found between the self-report Sleep Inertia Questionnaire (SIQ) and the Composite Scale of Morningness [20]. It is, therefore, possible that evening types, being disadvantaged by social schedules (more favorable to morning types), are more easily sleep deprived and may, therefore, experience greater sleep inertia than morning types. Indeed, when individuals are free to sleep, the relationship between eveningness and sleep inertia disappears [21].

The present work aimed to clarify the relationship between circadian preference and motor wake inertia and motor sleep inertia using the actigraphic methodology. In particular, we aimed to evaluate motor wake inertia and motor sleep inertia in a group of morning- and evening-type adolescents. If the morning affect construct is comparable to that of the sleep inertia phenomenon, we would expect to find greater motor sleep inertia in evening-type participants. Conversely, if the two constructs are independent, we would expect to find no significant differences between the two extreme chronotypes. Since there are no similar works in the literature, it was difficult to make specific predictions about motor wake inertia. As for morning and evening type stereotypes, we may expect greater motor wake inertia in evening-type participants. However, a recent work analyzing sleep features in extreme chronotypes in adolescents using actigraphy found no differences in sleep onset latency [22].

## 2. Materials and Methods

### 2.1. Participants

**Figure 1:**
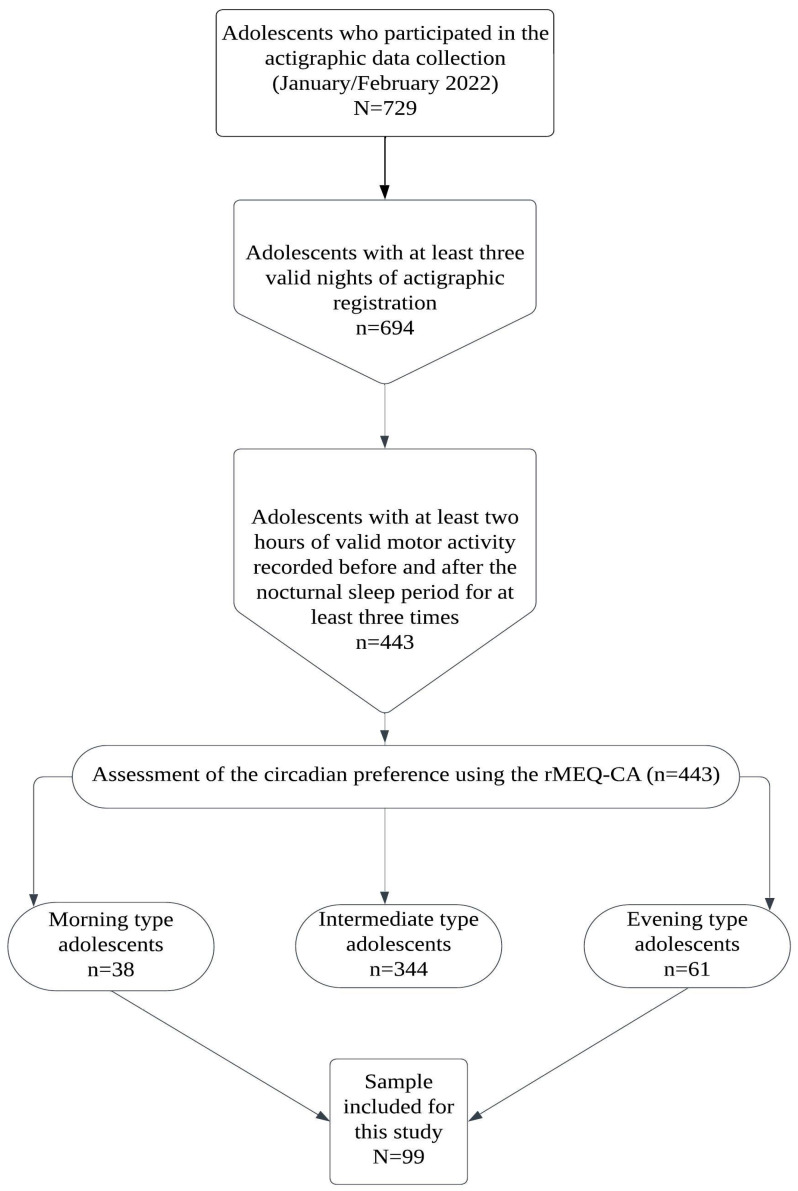
Figure 1 shows the experiment diagram explaining the participant selection for the present research.

Participants for this study are drawn from the project IDENTITIES. For the current study, 729 adolescents were considered. Among them, 443 adolescents wore an actigraph for at least two hours of valid motor activity recorded before and after the nocturnal sleep period and at least three times. According to the reduced Morningness–Eveningness Questionnaire for Children and Adolescents (rMEQ-CA) cut-off scores, 61 participants (339 nights considered) belonged to the evening-type category while 38 (235 nights considered) belonged to the morning type. An overall number of 99 participants (62.6% females), with a mean age of 15.6 years (standard deviation = 1.2 years, range = 13–18 years), were hence examined in the current study (See Figure 1).

Participants were either in the first (49.5%) or the third (50.5%) year of secondary education. More than half of the adolescents were in high school (57.6%), and the remainder in a technical (31.3%) or professional institute (11.1%).

### 2.2. Instruments

**Circadian preference**. The circadian preference was assessed using the Italian version [22] of the rMEQ-CA. The rMEQ-CA comprises five items—two multiple choice and three open—extracted from the MEQ-CA [23]. Participants are asked to list their preferred time to get up, when they usually go to bed, whether they feel tired after waking up in the morning, when they reach their “best peak” during the day, and whether they identify as “morning” or “evening” profiles. The total score, ranging from 4 to 25, is produced by adding the scores of each item. Morning types fall into the range of 19 to 25, intermediate types fall between 11 and 18, and evening types fall between 4 and 10 on the overall score [22]. Excellent external validity is demonstrated by the questionnaire [22].

**Motor activity**. In order to evaluate circadian motor activity, adolescents were asked to wear a wrist actigraph (Micro Motionlogger Watch, Ambulatory Monitoring, Inc.; Ardsley, NY, USA) 24 h per day for a whole week. The hardware consists of a piezoelectric accelerometer with a sensitivity ≥0.01 g. The sampling frequency is 10 Hz, and filters are set to 2–3 Hz. The actigraph was initialized through the Motionlogger Watchware software (Ambulatory Monitoring, Inc., Ardsley, NY, USA) in zero-crossing mode to collect data in 1 min epochs. Adolescents were instructed to wear the actigraph for one week and to push the event marker button on the actigraph to indicate when they (a) turned off the lights to go to sleep at night, and (b) got out of bed in the morning. The actigraphic data were analyzed through Action W-2^®^ software, version 2.7.1 (Ambulatory Monitoring, Inc., Ardsley, NY, USA). This software identified each epoch as sleep or wake using the mathematical model validated by Cole and Kripke [24] and Cole and colleagues [25]. This mathematical model computed a weighted sum of the activity in the current epoch, the preceding 4 epochs, and the following 2 epochs as follows: S = 0.0033 (1.06an4 + 0.54an3 + 0.58an2 + 0.76an1 + 2.3a0 + 0.74a1 + 0.67a2), where an4 to an1 were the activity counts from the previous 4 min, and a1 and a2 were those related to the following 2 min. The current minute was scored as sleep when S < 1. Action W-2 underwent 5 additional re-scoring rules [26] developed to minimize the tendency of actigraphy to overestimate total sleep time. Participants were instructed to push the event-marker button on the device to mark occurrences such as time in and out of bed. During the recorded period, subjects were also asked to fill in the sleep log daily within 30 min of morning awakening. In this way, it was possible to identify the bedtime and the wake-up time even if the event mark button had not been pressed.

In the present study, we considered two actigraphic sleep parameters: bedtime and get-up time. The Action 4 software (Ambulatory Monitoring, Inc., Ardsley, NY, USA) was used to extract the raw motor activity counts, minute-by minute, in order to draw motor activity patterns around the wake–sleep and sleep–wake transition phases. In particular, we explored the raw motor activity pattern of the wake–sleep transition for each recorded day by extracting the motor activity counts from 120 min before bedtime up to 60 min after bedtime on school days only, and the raw motor activity pattern of the sleep–wake transition for each recorded day by extracting the motor activity counts from 60 min before get-up time up to 120 min after get-up time.

### 2.3. Procedure

As part of the ERC-Consolidator project IDENTITIES, “Managing identities in diverse societies: A developmental intergroup perspective with adolescents,” the bioethics committee of Alma Mater Studiorum—University of Bologna (Bologna, Italy) approved the current study (Prot. no. 263836 of 14/10/2021). This study is a component of a longer longitudinal research project that involved instructors, parents, and first- and third-year students (at the initial assessment) from various high schools in the Emilia-Romagna region of northern Italy.

Principals were presented with the initiative after schools were chosen using a stratified random process based on school tracking and the degree of urbanization in the area. Parents and students, who also received written and comprehensive material, were then introduced to the study procedure. For students under the age of eighteen, parental active consent was gained; for those over eighteen, consent was obtained directly. Students were made aware that their participation in the study was completely voluntary and that they might revoke their agreement at any moment.

Beginning in 2022, the ongoing longitudinal project entails several annual, monthly, and daily examinations. The current study analyzed data from the first wave, which occurred in January and February of 2022. Only those who had at least three valid nights in the actigraphic recording and who had accurately completed the questionnaire to determine circadian preference were included in the final database for statistical analysis. Questionnaires on circadian preference and other constructs (such identity development) were given out during school hours following the actigraphic evaluation.

### 2.4. Statistical Analyses

In order to investigate variation in the motor activity pattern of the wake–sleep and sleep–wake transition, according to the circadian preference, functional linear modeling (FLM) [27] was utilized. In the Functional Linear Modeling (FLM) context, data processing primarily involves transforming discrete observations into functional representations and selecting an appropriate basis for expansion, such as the Fourier series. This Fourier expansion enables data representation through sinusoidal functions, capturing the main features of periodic or quasi-periodic data with a reduced number of coefficients. This approach enhances both computational efficiency and interpretability within the FLM framework. The Fourier basis transforms the data into a manageable set of coefficients, facilitating analysis in the FLM by reducing dimensionality and capturing smooth functional patterns. A 180 min time window was extracted for FLM analysis in order to examine wake inertia. Specifically, a 120 min interval was taken prior to bedtime and a 60 min interval was taken after bedtime. For sleep inertia, a 180 min time window was taken as well, with an interval of 60 min before and 120 min after get-up time. Groups of participants were then divided into different groups based on their chronotype for the statistical analyses. First, the Fourier expansion model was used to convert the raw motor activity pattern into a functional form. Second, within the previously specified time intervals, the non-parametric permutation F-test was utilized to ascertain whether and when the motor activity pattern of the wake–sleep and sleep–wake transition underwent a significant change based on circadian preference. This test employs random reassignment of data labels to generate an empirical distribution of the F-statistic, against which the observed value is compared. This approach circumvents rigid distributional assumptions, providing robust statistical conclusions even for complex data structures. The “Actigraphy” package in the R statistical program was used to carry out the FLM.

## 3. Results

**Figure 2:**
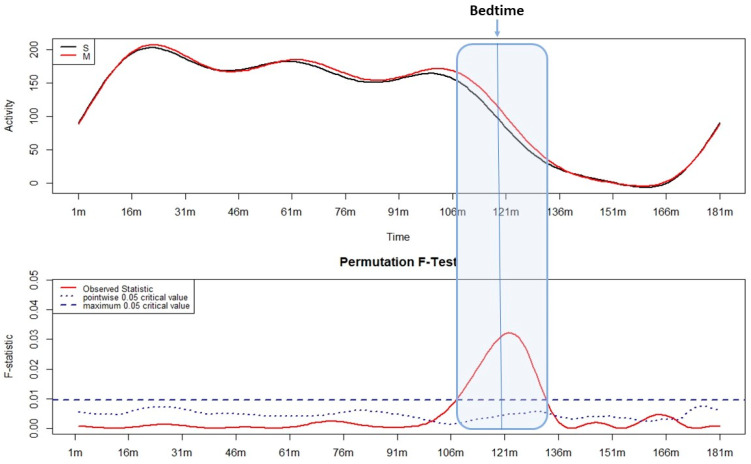
Results of the functional linear modeling applied to motor activity patterns during the wake–sleep transition based on circadian preference. The upper panel illustrates the variation in motor activity of morning (in red) and evening (in black) types from 120 min before to 60 min after bedtime (motor activity level is shown on the y-axis; the time is shown on the x-axis in minutes). In the lower panel, the statistical results (non-parametric permutation F-test) are displayed: when the solid red line exceeds the dashed blue line, the differences shown in the upper panel are considered statistically significant (F value is shown on the y-axis; the time is shown on the x-axis in minutes).

Results related to motor wake inertia according to circadian preference are shown in Figure 2. The differences in the motor activity pattern reached statistical significance both before and after bedtime, in particular from 15 min before bedtime until 12 min after bedtime. In this time window, a significantly higher level of motor activity was observed in morning-type participants compared with evening-type participants.

**Figure 3:**
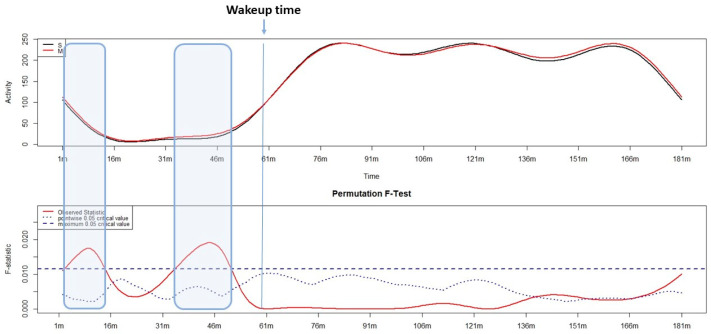
Results of the functional linear modeling applied to examine the variation in motor activity during the sleep–wake transition according to circadian preference. In particular, the upper panel shows the variation in motor activity of morning- (in red) and evening- (in black) type participants from 60 min before to 90 min after the wake-up time (motor activity level is shown on the y-axis; the time is shown on the x-axis in minutes). The lower panel shows the statistical results (non-parametric permutation F-test) with significant differences when the solid red line (the observed statistics) is above the dashed blue line (motor activity level is shown on the y-axis; the time is shown on the x-axis in minutes).

The variation in the motor activity pattern of the sleep–wake transition according to circadian preference are shown in Figure 3. In this case, we find a statistical significance at two time points, both before the morning awakening: the first, lasting around 10 min, one hour before the morning awakening; and the second, lasting around 14 min, half an hour before the morning awakening. In both windows, a significantly higher level of motor activity was observed in morning-type participants compared to evening-type participants. No significant difference was observed after waking up in the morning.

## 4. Discussion

Regarding motor wake inertia, the results showed a significant difference in the moment at which the participants turned off the light to try to fall sleep. In particular, the morning types showed significantly greater motor activity than the evening types for about 10 min before bedtime until about 15 min after. This result may seem counterintuitive, since morning people are the first to feel the need to sleep in the evening and consequently go to sleep earlier. This apparent paradox could be due precisely to the fact that our evening sleepers, by procrastinating regarding the time they go to sleep, had accumulated a greater need for sleep when they go to bed, which could affect and lower their levels of motor activity. To verify this aspect, future research that involves manipulating the time in bed will be necessary. However, since we only considered the days when adolescents went to school, differences in time in bed due to circadian preference could have been attenuated. Our results would therefore seem to indicate that morning types experience greater wake inertia than evening types. Based on what is reported in the literature, this characteristic does not seem to have an effective impact, for example, on the latency to fall asleep in healthy participants [22,28]. However, this is another aspect that should be further explored.

Regarding motor sleep inertia, a statistically significant difference was found only before the moment of awakening. In particular, the morning types showed greater motor activity than the evening types at two times: the first about an hour before waking up with a duration of about 10 min; and the second about half an hour before waking up with a duration of about 15 min. In this case, the wake-up time effect could have been attenuated by the fact that, having only considered the days on which the adolescents went to school, the wake-up time was dictated more by social synchronizers than by individual preferences. These data seem to indicate that the extreme chronotypes differ in the awakening phase, but that this difference appears significant before awakening. No significant differences were observed between the chronotypes after the moment of awakening. This result seems to suggest that the difference between extreme chronotypes at the moment of awakening labeled as morning affect can also be documented with actigraphy, but that is something different from sleep inertia. Clearly, further studies will be necessary to clarify this important aspect.

Considering these results as a whole, it would seem possible to conclude that morning types have more difficulty in transitioning from the waking condition to the sleeping condition, while upon awakening they are facilitated in transitioning from the sleeping condition to the waking condition by a sort of greater activation that precedes the moment of awakening [29]. In sum, it is as if morning types are more inclined to be awake, while evening types are more inclined to be asleep. This reflection appears particularly interesting especially if we refer to the wake–sleep cycle model of regulation called the two-process model [30]. This model posits that sleep is regulated by the interaction of a sleep–wake-dependent homeostatic process (Process S) with a process controlled by the circadian pacemaker (Process C). Therefore, we could speculate that the differences between morning and evening types derive from a different interaction between the two processes: morning types are more inclined to follow the time dictated by the C process, while evening types are more affected by the S process. The paradigm that considers circadian preference as an independent variable was proposed as an ecological paradigm to distinguish the role of S and C processes [31]. Future research could further examine this fascinating hypothesis.

## 5. Conclusions

In this work, we demonstrated the usefulness of actigraphy applied in an innovative way. The advantage of actigraphy is that it can collect a large number of data in a purely ecological context. In particular, we have attempted to understand the difference between morning affect and sleep inertia. With these results, it would seem possible to conclude that sleep inertia and morning affect are two different aspects of behavior. The results are interesting and promising, but need to be tested further, for example in populations of diverse ages.

## Data Availability

The original data presented in the study are openly available in Open Science Framework (OSF), page of the project: https://osf.io/njsqr/.
